# Efficacy and safety of an extended-release sebacoyl dinalbuphine ester for laparoscopic cholecystectomy: A randomized controlled trial

**DOI:** 10.1097/MD.0000000000034423

**Published:** 2023-08-04

**Authors:** Ying-En Lee, Chen-Yu Fu, Yow-Ling Shiue, Chu-Yun Lu, Chung-Yen Chen, Jian-Han Chen, Jen-Lung Chen, Chen-Fuh Lam

**Affiliations:** a Department of Anesthesiology, E-Da Hospital and I-Shou University, Kaohsiung, Taiwan; b Institute of Biomedical Sciences, College of Medicine, National Sun Yat-Sen University, Kaohsiung, Taiwan; c Department of Anesthesiology, Shin-Huey-Shin Hospital, Kaohsiung, Taiwan; d Department of Nursing, I-Shou University College of Medicine, Kaohsiung, Taiwan; e Institute of Precision Medicine, College of Medicine, National Sun Yat-Sen University, Kaohsiung, Taiwan; f Division of General Surgery, Department of Surgery, E-Da Hospital and I-Shou University, Kaohsiung, Taiwan; g School of Medicine, I-Shou University College of Medicine, Kaohsiung, Taiwan; h Bariatric and Metabolism International Surgery Center, E-Da Hospital and I-Shou University, Kaohsiung, Taiwan; i Department of Anesthesiology, Dalin Tzu Chi Hospital, Buddhist Tzu Chi Medical Foundation, Chia-Yi, Taiwan.

**Keywords:** chronic postsurgical pain, drug adverse reaction, laparoscopic cholecystectomy, multimodal analgesia, parenteral analgesic, SDE

## Abstract

A long-acting κreceptor agonist parenteral analgesic may theoretically improve acute pain and reduce incidence of chronic postsurgical pain (CPSP) after laparoscopic cholecystectomy with minimal drug-related side effects of the traditional μreceptor opioids.

**Methods:**

Eighty adult patients undergoing elective laparoscopic cholecystectomy were randomly assigned to receive single intramuscular injection of an extended-release sebacoyl dinalbuphine ester (SDE, Naldebain 150 mg; *n* = 40) or placebo (*n* = 40) after anesthesia induction. Standard multimodal analgesia (MMA) was administered for postoperative pain control. The primary endpoint was pain intensity within 7 days after surgery. The secondary endpoints were incidence CPSP at 3 months and adverse reactions up to 7 days after surgery.

**Results:**

The highest visual analogue scale (VAS) and area under the curve of VAS 0 to 48 hours after operation were not different between the two groups and a similar proportion of patients requested rescue parenteral analgesics. Average pain intensities were also not different at 72 hours and 7 days after surgery. Incidence of CPSP was 22.5% and 13.1% in patients who received placebo and SDE treatment, respectively (*P* = .379). Significantly higher incidence of drug-related adverse events, including dizziness, nausea and injection site reactions, were recorded in the SDE group.

**Conclusion:**

A single dose of extended-release analgesic SDE given intraoperatively did not provide sufficient add-on effect for acute and chronic pain management after laparoscopic cholecystectomies in patients who received standard postoperative MMA. Intramuscular injection of 150 mg SDE in patients with average body mass causes adverse events that could have been overlooked. More clinical studies are warranted to determine the target populations who may benefit from SDE injections for improvement of acute and chronic postsurgical pain management.

## 1. Introduction

Cholecystectomies are a common procedure for the surgical removal of inflammed or otherwise pathological gall bladders and most cholecystectomies are performed laparoscopically in Taiwan (>85%).^[1]^ Patients may still suffer from acute postoperative surgical-related pain even with the minimally invasive laparoscopic operations. It has been found that up to 80% of patients undergoing laparoscopic cholecystectomies complained of moderate-to-severe pain within the first day after the operation.^[2]^ Most importantly, 20% of the patients may develop persisting chronic postsurgical pain (CPSP) within 1 year postoperatively and they require prolonged use of opioid analgesics to control the CPSP.^[3]^ Several important clinical observational studies have suggested that inadequate control of surgical-related pain at the initial week after cholecystectomy is one of the major risk factors for developing CPSP.^[4]^ Since the length of hospital stays after uncomplicated laparoscopic cholecystectomies are relatively short (usually within 24 hours post-operatively),^[5,6]^ postoperative pain is inevitably managed by repeated doses of oral form analgesics, including opioids.^[2]^ Management of postoperative pain using a multimodal analgesia (MMA) approach is widely recommended by the international scientific societies by administration of drugs with differing mechanisms of actions target pain pathways resulting in additive and/or synergistic effects to opioids.^[2]^ Therefore, a long-acting parental analgesic in addition to the standard MMA could be a viable strategy to achieve optimal control of acute postoperative pain after laparoscopic cholecystectomies in patients who are discharged early (within 24 hours). This strategy could also reduce the incidence of CPSP as plasma therapeutic levels of the parental analgesic can be maintained for several days after injection.^[7]^

Nalbuphine is a semi-synthetic opioid that acts as a mixed κreceptor agonist and μreceptor antagonist, but its clinical applications for acute postoperative pain is limited by its relatively short duration of action of 3 to 6 hours.^[8]^ Sebacoyl dinalbuphine ester (SDE, Naldebain®) is the prodrug of nalbuphine, and was approved by the Taiwan FDA in 2017.^[9]^ After intramuscular injection, SDE is hydrolyzed by tissue esterase to release the active compound nalbuphine. The systemic bioavailability of nalbuphine is 85.4% with a mean absorption time of up to 145 hours.^[10]^ Therefore, a single parenteral injection of SDE may provide a prolonged analgesic effect for 6 days. Currently, there have been 3 prospective randomized double-blind trials investigating the efficacy and safety of SDE for pain management after hemorrhoidectomy,^[11]^ laparotomy^[12]^ and laparoscopic bariatric surgery.^[13]^ The clinical outcomes of these previous studies show that SDE is superior to the conventional or patient-controlled analgesia for the management of acute postoperative pain with less need for rescue analgesics and more tolerable adverse drug reactions. However, patients who were assigned to control groups in the first 2 studies did not receive the standard MMA treatment, and periods of observation for adverse events were not specified in the study protocols. Furthermore, SDE was administered at least 12 hours before surgical intervention in these studies, which can be a major limitation for the application of SDE in the day-case surgeries, such as laparoscopic cholecystectomy.^[6]^ Therefore, this clinical study was designed to determine the extended analgesic effect of SDE administered after anesthesia induction as a component of MMA for laparoscopic cholecystectomy and evaluate the drug-related adverse events up to 7 days after administration.

## 2. Methods

### 2.1. Setting and participants

This phase II/III randomized, double-blind, placebo-controlled clinical trial was conducted in E-Da Hospital and E-Da Cancer Hospital (Kaohsiung, Taiwan) from August 2021 to April 2022. Adult patients aged 20 to 65 years with American Society for Anesthesiologist physical statuses ≤ III who were scheduled for laparoscopic cholecystectomies. This study included patients who were anticipated to stay in the hospital for > 72 hours after operation for feasibility to observe the analgesic effect and potential drug-related adverse events following administration of study drugs. The study was approved by the institutional review board of the E-Da Hospital, Kaohsiung, Taiwan (approval number EMRP28110N). The trial was registered in March 2021 with Clinicaltrials.gov registration: NCT04808544. All participants provided written informed consent before the commencement of the study.

### 2.2. Exclusion criteria

Patients who were scheduled for laparotomies or urgent operations and patients with chronic opioid use, with allergies to Nalbuphine, benzyl benzoate, or sesame oil were excluded from the study. Patients who might require ventilator support after surgery and those who were not able for assessment of pain intensity or refused to participate in questionnaire surveys were also excluded.

### 2.3. Allocation to intervention

Participants were randomly assigned to receive SDE (150 mg in 2 mL vehicle; Lumosa Therapeutics Co., Ltd., Hsinchu, Taiwan) or vehicle solution (2 mL of mixture of benzyl benzoate and sesame oil) in permuted blocks of 10 using a computer-generated list. Dose of SDE used in this study was derived from the previous pharmacokinetic study^[10]^ and other clinical trials.^[11–13]^ Patient’s treatment allocation was concealed in an opaque envelope and the envelope was opened after the patient was admitted to the holding area of the operating room by the research assistant.

### 2.4. Intervention protocols

SDE (nalbuphine in solvent containing benzyl benzoate and sesame oil) and the vehicle solution (solvent containing benzyl benzoate and sesame oil) was prepared in an identical injection syringe within 30 minutes before injection. The research drugs were prepared by a research nurse who did not involve in the clinical care. The surgical procedure was performed under endotracheal intubation general anesthesia and controlled ventilation. Anesthesia was maintained by inhalational anesthetics (desflurane or sevoflurane) at optimal minimal alveolar concentrations and bispectral index levels. After anesthesia induction, SDE or the vehicle solution was injected into the gluteus muscles by the in-charge anesthesiologist under ultrasound guidance. Since study drugs were prepared by the research nurse, the patients, anesthesiologists, surgeons, and research team members who assessed the study outcomes were blinded to the treatment groups. Dexamethasone (5 mg) was given intravenously after induction of anesthesia and intravenous Palonosetron (0.25 mg) was administered in patients who had high risk of developing postoperative nausea and vomiting. Parecoxib (40 mg) and propacetamol (2 g) were administered intravenously at the end of the operation as the standard postoperative MMA.^[2,14]^ Parenteral opioids, including morphine and fentanyl, were individually prescribed by the anesthesiologist according to the patient’s hemodynamic changes and nociceptive responses during operation. At the end of operation, the endotracheal tube was extubated in the operating room. Patients were transferred back to the original wards from the post-anesthetic care unit. Patients were commenced on regular oral analgesics after resumption of normal oral intake, including acetaminophen and non-steroidal anti-inflammatory drugs (NSAID). Rescue parenteral analgesics, including opioids, parecoxib and NSAIDs were prescribed by the anesthesiologists or surgeons when visual analog scale (VAS) remained > 4 after regular oral analgesics were administered. The intraoperative MMA regimen, including SDE, parecoxib and propacetamol were supported by the research funds. The vehicle solutions were generously provided by the Lumosa Therapeutics Co. Ltd. (Hsinchu, Taiwan).

### 2.5. Study endpoints and measurements

The primary endpoint of this study was the intensity of surgical pain from post-anesthetic care unit to 7 days postoperatively. Pain intensity was assessed by ward nurses using the visual analog scale (VAS, 0 represents “no pain” and 10 represents “worst pain”) in 4-hours intervals from 0 to 48 hours after operation. The numeric rating scale (0 represents “no pain” and 10 represents “worst pain”) was used to evaluate the pain intensity at 72 hours and 7 days postoperatively by the outcome assessors who did not participate in the clinical care. Acute postsurgical pain was defined as unpleasant sensation related to tissue injury during the acute phase after surgical intervention.^[15]^ Areas under the curve (AUC) of the VAS were calculated by plotting the individual VAS score as curves in which the x-axis represented measurement time points from baseline (4-hours intervals) to 48 hours after surgery, and the y-axis represented the VAS score.^[16]^ The secondary endpoints included total doses of rescue parenteral analgesics administered during hospital stay period, length of stay after operation, incidence of CPSP and patient’s quality of life (QoL). CPSP was defined as the newly developed pain at the surgical site or incisional wounds within 3 months after surgery, as other etiologies of pain were excluded.^[17]^ QoL was evaluated by the 12-item short form (SF-12) questionnaire^[18]^ on hospital admission, and at 7 and 90 days after surgery. The scores of SF-12 questionnaire range from 0 to 100, and lower scores indicate more impaired health-related QoL. Systemic and focal drug-related adverse reactions were recorded up to 7 days after surgery.

### 2.6. Data analysis

A recent study showed that SDE significantly reduced the VAS by 32% at 48 hours after elective laparotomy surgery.^[12]^ In comparison to laparotomies, the intensity of postoperative pain is generally reduced in laparoscopic procedures. Therefore, our study proposed that SDE injection might reduce surgical related pain by 20% after laparoscopic cholecystectomies. With a pre-defined 20% standard error, a total sample size of 80 patients (including a dropout rate of 10%) was desirable to detect a 20% difference in pain reduction with an α value 0.05 and statistical power 0.8. Categorical variables were compared using the Fisher’s exact test. Comparisons of the continuous variables between the 2 study groups were analyzed using the Student *t* test or Mann–Whitney *U* test, as appropriate. Two-way repeated measures ANOVA was used to compare SF-12 scores at baseline and after surgery. All analyses were carried out using the SAS software, version 9.1 (SPSS software, version 24.0; IBM, Armonk, NY).

## 3. Results

### 3.1. General outcomes

**Figure 1.**
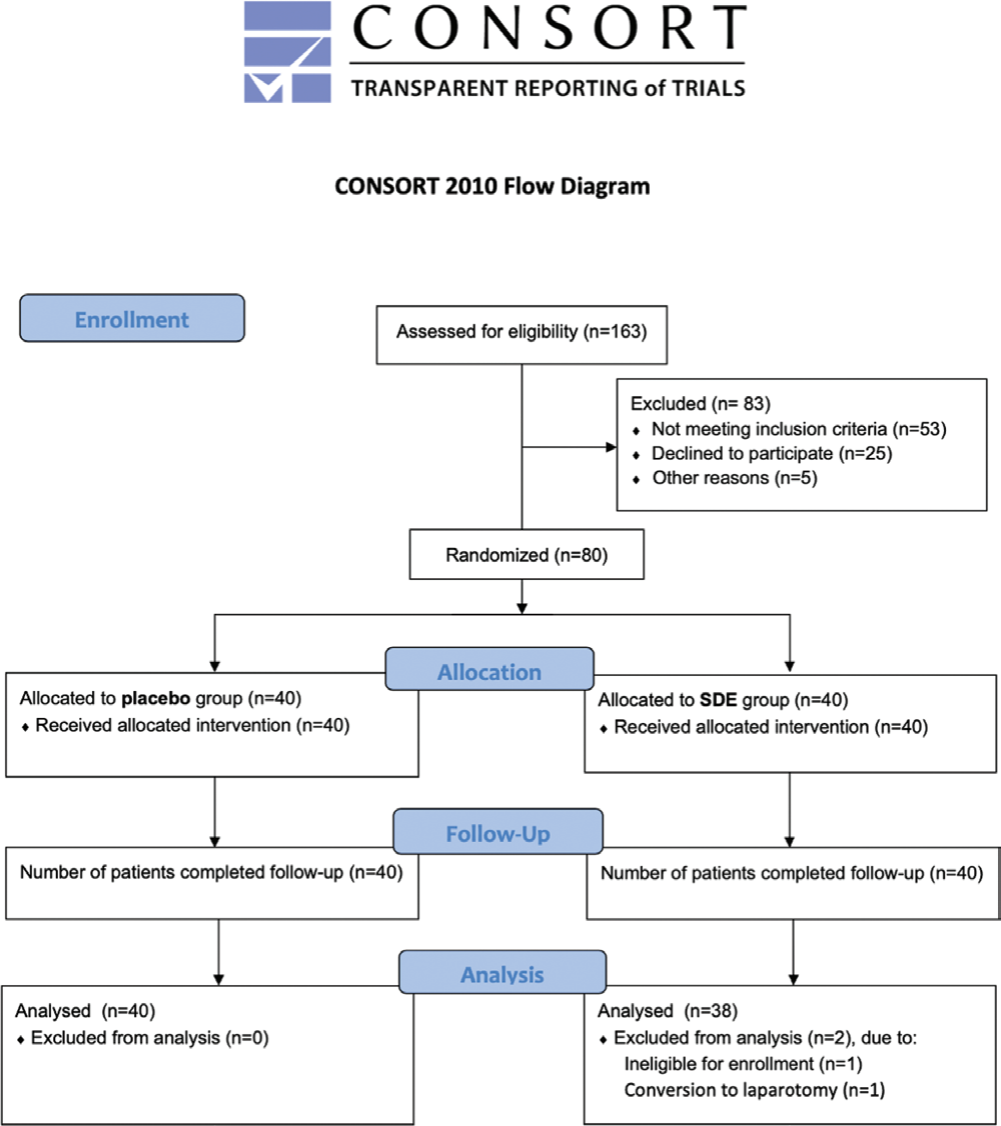
The CONSORT study flow diagram. SDE = sebacoyl dinalbuphine ester.

During the study period, a total of 163 patients were evaluated for inclusion and 83 patients were excluded due to ineligibility and patient refusal (Fig. 1). 80 patients (n = 40 each) were randomly assigned to the 2 study groups before anesthesia induction (Fig. 1). Two participants in the SDE group were excluded from the final analysis due to unintentional enrollment of a patient who was over 65 years old and a patient who was converted to laparotomy after failure of the laparoscopic approach during the operation (Fig. 1). Patient demographic data were not different between the 2 study groups (Table 1). The lengths of hospital stay after operation were 2.6 ± 1.4 days and 2.7 ± 1.4 days in the placebo and SDE groups, respectively (Table 1). Intravenous morphine was administered during operation in 15% and 18.4% of the patients for postoperative pain control in the placebo and SDE groups, respectively (Table 1).

**Table 1:** Basic patient demographic data.

	SDE (n = 38)	Placebo (n = 40)
Age (yr)	46.0 ± 10.9	48.2 ± 10.4
Female	28	21
Body mass index (kg/m^2^)	26.3 ± 4.9	26.7 ± 4.4
ASA physical status (I:II:III)	4:33:1	8:31:1
Active smoker	7	9
History of PONV	7	6
Length of hospital stay (d)	2.7 ± 1.4	2.6 ± 1.4
Intraoperative morphine	7	6

Data are presented as n or mean ± SD.

ASA = American Society of Anesthesiologists, PONV = postoperative nausea and vomiting, SDE = sebacoyl dinalbuphine ester.

### 3.2. Primary endpoint

The AUC for the VAS from 0 to 48 hours after surgery were 119.4 ± 41.6 and 116.0 ± 42.6 in the placebo and SDE groups, respectively (*P* = .395) (Table 2). The highest VAS within 48 hours after cholecystectomy were also similar between 2 groups (4.0 ± 1.7 vs 4.0 ± 1.5 for placebo vs SDE; *P* = .893) (Table 2). The pain intensities were generally reduced after 48 hours, and the mean scores of numeric rating scale were also not different between the 2 groups at postoperative 72 hours and day 7 (Table 2).

**Table 2:** Primary and secondary endpoints.

	SDE (n = 38)	Placebo (n = 40)	*P* value
VAS AUC (0–48 h)	116.0 ± 42.6	119.4 ± 41.6	.395
VAS AUC (0–24 h)	66.0 ± 24.1	67.6 ± 23.3	.499
VAS AUC (24–48 h)	50.1 ± 19.3	51.8 ± 19.4	.514
Highest VAS (0–48 h)	4.0 ± 1.5	4.0 ± 1.7	.893
Average VAS (0–48 h)	2.3 ± 0.7	2.6 ± 0.8	.144
NRS at 72 h	2.1 ± 1.3	2.0 ± 1.1	.912
NRS at 7 d	1.2 ± 1.2	1.0 ± 0.9	.769
Rescue analgesics			
0–24 h	26 (68.4)	29 (72.5)	.805
24–48 h	3 (7.9)	0	.111
CPSP at 90 d	5 (13.1)	9 (22.5)	.379

Data were analyzed using independent *t* test or Fisher exact test, as appropriate. Results are presented as n (%) or mean ± SD.

AUC = area under the curve, CPSP = chronic postsurgical pain, NRS = numerical rating scale, SDE = sebacoyl dinalbuphine ester, VAS = visual analog scale.

### 3.3. Secondary endpoints

**Figure 2.**
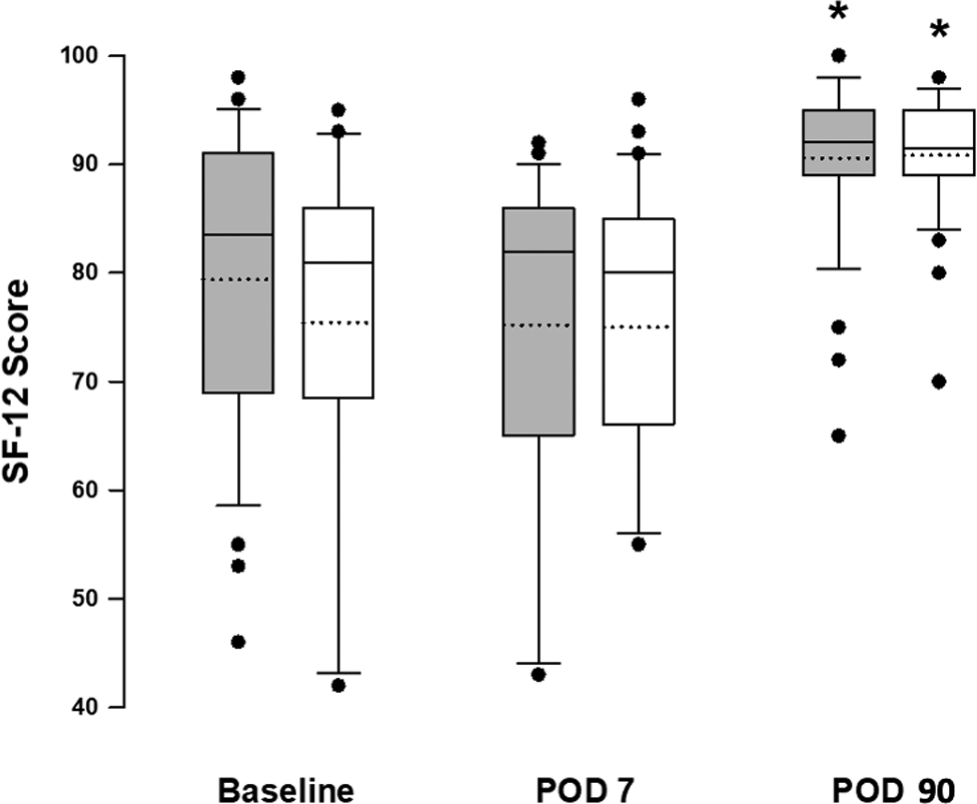
QoL assessed by the SF-12 questionnaire before operation, and at POD 7 and 90. The SF-12 scores of the SDE (filled column) and placebo (open column) groups measured at different time points were compared using the 2-way repeat measures ANOVA, followed by the Bonferroni test for post hoc analysis. Results are presented as box-and-whisker plots, in which the solid horizontal lines of boxes indicate the 75th percentile, median and 25th percentile of the distribution, the upper and lower whiskers indicate the maximal and minimal values, and the dotted horizontal lines indicate mean values. *P < .05 vs the baseline values within each group. POD = postoperative day, QoL = quality of life, SDE = sebacoyl dinalbuphine ester.

From 0 to 24 hours after operation, rescue parenteral analgesics were required in 29 (72.5%) and 26 (68.4%) patients in the placebo and SDE groups, respectively (*P* = .805), and fewer patients required rescue analgesics from 24 to 48 hours postoperatively in both groups (0 vs 3, *P* = .111) (Table 2). The incidence of CPSP at 3 months after laparoscopic cholecystectomy was 22.5% (9 in 40) in the placebo group and 13.1% (5 in 38) in the SDE group (*P* = .379) (Table 2). The mean scores of SF-12 were generally lower in patients who had indications for surgical removal of gall bladder preoperatively and at 7 days after laparoscopic cholecystectomy, but QoL significantly improved compared to the average scores of > 90 at 90 days post-operatively in both groups (Fig. 2). The SF-12 scores did not differ significantly between the placebo and SDE groups at the 3 measurement time points (Fig. 2).

### 3.4. Drug-related adverse reactions

The potential systemic and focal adverse reactions following intramuscular injection of the study drugs were recorded up to 7 days after surgery (Table 3). No cases of pyrexia (body temperature > 37ºC) were reported in both groups. A significantly higher incidence of dizziness (defined as feeling faint, weak or/and unsteady gait^[19]^) and nausea (defined as feeling of retching or stomach distress with an urge to vomit^[19]^) was found in patients who received SDE within 72 hours post-operatively (Table 3). The SDE group was also associated with a significantly higher incidence of injection site reactions and soreness, particularly at 72 hours and 7 days after local injection (Table 3). However, medical intervention for injection site problems was not required throughout the study period.

**Table 3:** Drug-related adverse effects.

	SDE (n = 38)	Placebo (n = 40)	*P* value
Pyrexia (>37ºC)	0	0	–
Dizziness			
0–24 h	23 (60.5)	14 (35.0)	.040
At 48 h	19 (50.0)	5 (12.5)	.0005
At 72 h	15 (39.5)	2 (5.0)	.0002
At 7 d	3 (7.9)	0	.111
Nausea (±vomiting)			
0–24 h	18 (47.4)	15 (37.5)	.492
At 48 h	9 (23.7)	2 (5.0)	.023
At 72 h	5 (13.1)	0	.024
At 7 d	0	0	–
Injection site soreness*			
0–24 h	4 (10.5)	3 (7.5)	.708
At 48 h	10 (26.3)	4 (10.0)	.079
At 72 h	18 (47.4)	5 (12.5)	.001
At 7 d	13 (34.2)	1 (2.5)	.0002
Injection site reactions†			
0–24 h	0	0	–
At 48 h	4 (10.5)	1 (2.5)	.195
At 72 h	9 (23.7)	1 (2.5)	.006
At 7 d	8 (21.0)	2 (5.0)	.045

Data were analyzed using the Fisher exact test and are presented as n (%). Dizziness was defined as feeling faint, weak or/and unsteady gait.^[19]^ Nausea was defined as feeling of retching or stomach distress with an urge to vomit.^[19]^

SDE = sebacoyl dinalbuphine ester.

* Tenderness or painful sensation on the injection site of SDE or placebo solution.

† Reactions including erythematous change and focal swelling.

## 4. Discussion

Three previous randomized double-blind placebo-controlled trials have demonstrated that a single preoperative injection of 150 mg SDE provides significant clinical analgesic effects in the management of post-open surgery pain.^[11–13]^ However, the results of this clinical trial did not find any significant additive analgesic effect of SDE compared with the intraoperative MMA only group (parecoxib and propacetamol with or without morphine) for acute pain up to 7 days after laparoscopic cholecystectomy. In comparison to the previous controlled studies,^[11–13]^ there were 2 main differences in our study design. First, the study drug was injected after induction of anesthesia in this study rather than administered > 12 hours preoperatively. Administration of SDE after anesthesia induction may not only reduce the discomfort of deep intramuscular injection in conscious patients, but also allows the treatment for day-case surgeries or when preoperative admission is not mandatory. Although the onset of SDE therapeutic effects could be delayed for 12 hours, this study did not detect clinically significant beneficial responses in pain intensity reduction from 24 hours to 7 days after administration. Secondly, standard parenteral MMA was administered intraoperatively to all participants in this study and the previous trial reported by our research team,^[13]^ whereas the placebo groups in the 2 previous studies received only blank or suboptimal analgesics for postoperative pain control.^[11,12]^ Nevertheless, our study results are consistent with a previous open-label study, which demonstrated that preoperative administration of SDE did not significantly improve pain intensity and requirement for rescue analgesics after laparoscopic cholecystectomy.^[20]^ The results of these 2 studies suggest that laparoscopic cholecystectomies only induce mild-to-moderate levels of pain in the acute post-operative stage. The postoperative pain could be adequately managed by the standard opioid-sparing MMA regimen, including NSAID, selective cyclooxygenase-2 inhibitors, and acetaminophen.^[2]^

After intramuscular injection, SDE is hydrolyzed by tissue esterase and the active compound nalbuphine is constantly released over 145 hours to mediate a longer lasting analgesic effect.^[10]^ The presence of CPSP after laparoscopic cholecystectomy is known to increase the risk of chronic opioid use (odd ratio 1.62, 95% confidence interval 1.49–1.76).^[21]^ Therefore, the long-lasting analgesic effect of SDE could theoretically attenuate the development of CPSP.^[7]^ To our knowledge, this is the first study testing the potential beneficial effect of a long-lasting parenteral analgesic in the prevention of CPSP, as acute postoperative pain is more likely to transit into chronic pain if the acute pain is not adequately controlled and it persists beyond the usual tissue healing time of 1 week.^[21]^ Our results showed that the incidence of CPSP at 3 months after surgery in the placebo-controlled group was 22.5%, which is comparable with the findings of previous studies reported by other groups in the range of 3% to 59%.^[3,22,23]^ The incidence of CPSP was reduced by 41% in the SDE-treated group (22.5% vs 13.1%), but the difference was not statistically significant.

The drug-related adverse reactions of SDE were previously reported in the several prospective^[11,12,20]^ and retrospective^[24]^ clinical studies. In general, the adverse drug reactions include systemic responses reacting to the active compound nalbuphine (such as sedation, dizziness, nausea and vomiting) and focal reactions following intramuscular injection (such as soreness and tissue reactions at the injection site). These studies also noted an increase in unexpected pyrexia in patients who received SDE injection.^[11,20]^ It should also be noted that the plasma concentration of nalbuphine can be maintained at therapeutic levels for up to 6 days after administration, but onset and subsidence time frames of the drug-related adverse responses were not reported in these studies. In our study, the potential drug-related adverse events were recorded up to 7 days post-operatively. We did not record any cases of pyrexia while in hospital. Dizziness was the most common systemic adverse reaction in this study and was significantly higher in the SDE group through postoperative days 1 to 3. 39.5% of the patients in the SDE complained of dizziness on postoperative day 3 compared to only 5% in the placebo group. The incidence of nausea was similar between the placebo and SDE on the first day post-operatively, but significantly more patients in the SDE group remained nauseous on day 2 to day 3 postoperatively. At day 7, most of these systemic adverse events resolved spontaneously, except a small proportion (7.9%) of patients in the SDE group who reported ongoing dizziness. On the contrary, incidence and severity of SDE-related systemic adverse events were significantly reduced in overweight patients (body weight index > 38 kg/m^2^) who received laparoscopic bariatric surgery,^[13]^ suggesting that these adverse drug reactions might be dose-dependent and the therapeutic doses of SDE should be titrated according to the patient’s total body weight. Although injection site reaction seldom occurs following a single dose of intramuscular nalbuphine, our study and other study groups found that more than 20% of the patients receiving SDE injections experienced local erythema, swelling, and tenderness while only 6% to 12.5% of the patients who only received the vehicle solution injection developed focal reactions.^[11]^ In addition, we observed that injection site reactions (soreness and focal erythema/swelling) more often developed 48 hours after injections and could last for more than 5 days. However, these focal reactions at injection sites were usually mild and transient, and there were no participants who requested additional medical attention or management in this study. To avoid accidental administration into other subcutaneous tissues, such as the adipose layers, ultrasound-guided intramuscular injection of SDE is highly recommended.

This study also measured pre- and post-operative QoL using the SF-12 questionnaire, which includes physical and mental health composition domains. The average total SF-12 scores were found to be low both before and at 7 days post laparoscopic cholecystectomy in both groups (75–79). These scores increased significantly to > 90 at postoperative day 90, suggesting an improvement in physical and mental health after surgical removal of the pathological gall bladder. Since there were no differences in the SF-12 scores between the 2 groups at the 3 measurement time points, we suggest that the SDE-related adverse events were most likely tolerable and did not significantly affect the physical and mental recovery after laparoscopic cholecystectomy.

### 4.1. Study limitations

Several limitations of this study should be addressed. First, this study tested the postoperative analgesic effects of SDE that was administered after induction of anesthesia rather than > 12 hours before surgical intervention. According to the clinical pharmacokinetics of SDE, plasma therapeutic levels of nalbuphine are achieved at 12 hours after intramuscular injection.^[10]^ Therefore, the preemptive analgesic benefit of SDE might not be available if SDE was administered just before surgical stimulation. Second, all patients were interviewed over telephone at postoperative days 7 and 90 and the development of CPSP at postoperative day 90 was surveyed using a standard questionnaire. Since most of the patients who claimed to develop CPSP were unwilling to return to our pain clinic for further assessment, this study could only confirm that there was newly developed pain at the surgical sites or on the incisional wounds in some of the participants, but the severity and nature of the chronic pain were not assessed by pain specialists. Since postoperative MMA was administered in the 2 treatment groups, the effects of MMA on relieving of injection site reaction were not determined. Nevertheless, QoL was improved similarly in both groups at postoperative day 90, and none of these patients required medical attention for CPSP during the telephone interview. This suggests that the degree of chronic pain was mild, and it did not significantly affect post-operative recovery. Third, although there were no significant differences in the number of patients who requested for rescue parenteral analgesics up to 48 hours after surgery, differences in the total dosage and types of analgesics could exist between the 2 groups. Forth, although the incidence of CPSP was reduced in patients received SDE injection, this study was underpowered to detect a statistically significant effect of SDE to prevent the development of CPSP after laparoscopic cholecystectomy. Lastly, the drug-related adverse reactions could be dose-dependent. Further dose-ranging or divided dosing studies is advised to determine the clinical pharmacodynamics of SDE.

In conclusion, a single dose of the extended-release analgesic SDE administered intra-operatively did not provide a sufficient add-on effect to the standard MMA for acute and chronic postsurgical pain after laparoscopic cholecystectomy. On the contrary, intramuscular injection of 150 mg SDE in patients with average body mass can cause certain adverse events that may have been overlooked in previous reports. The adverse reaction profile and appropriate dosing of SDE should be more thoroughly investigated.

## Acknowledgments

The authors wish to appreciate Ms. Tzu-Shan Chen (Clinical Statistician, Department of Medical Research, E-Da Hospital and E-Da Cancer Hospital, Kaohsiung, Taiwan) for the advice in statistical analysis of the clinical data.

## Author contributions

**Conceptualization:** Ying-En Lee, Chen-Yu Fu, Jen-Lung Chen, Chen-Fuh Lam.

**Data curation:** Ying-En Lee, Chen-Yu Fu.

**Formal analysis:** Ying-En Lee, Chen-Yu Fu, Yow-Ling Shiue, Chu-Yun Lu, Jen-Lung Chen, Chen-Fuh Lam.

**Funding acquisition:** Chen-Fuh Lam.

**Investigation:** Ying-En Lee, Chen-Yu Fu, Chung-Yen Chen, Jian-Han Chen, Jen-Lung Chen, Chen-Fuh Lam.

**Methodology:** Ying-En Lee, Yow-Ling Shiue, Chu-Yun Lu, Jen-Lung Chen, Chen-Fuh Lam.

**Project administration:** Jen-Lung Chen, Chen-Fuh Lam.

**Resources:** Jen-Lung Chen, Chen-Fuh Lam.

**Software:** Yow-Ling Shiue, Chu-Yun Lu, Chen-Fuh Lam.

**Supervision:** Jen-Lung Chen, Chen-Fuh Lam.

**Visualization:** Jen-Lung Chen, Chen-Fuh Lam.

**Writing – original draft:** Ying-En Lee, Chen-Yu Fu, Chung-Yen Chen, Jian-Han Chen.

**Writing – review & editing:** Yow-Ling Shiue, Chu-Yun Lu, Jen-Lung Chen, Chen-Fuh Lam.
